# Comparing Response of Sheep and Cow Milk on Acute Digestive Comfort and Lactose Malabsorption: A Randomized Controlled Trial in Female Dairy Avoiders

**DOI:** 10.3389/fnut.2021.603816

**Published:** 2021-02-15

**Authors:** Aahana Shrestha, Linda M. Samuelsson, Pankaja Sharma, Li Day, David Cameron-Smith, Amber M. Milan

**Affiliations:** ^1^The Liggins Institute, The University of Auckland, Auckland, New Zealand; ^2^Riddet Institute, Palmerston North, New Zealand; ^3^AgResearch Ltd., Te Ohu Rangahau Kai, Palmerston North, New Zealand; ^4^Singapore Institute for Clinical Sciences, Agency for Science, Technology, and Research, Singapore, Singapore

**Keywords:** ovine milk, bovine milk, lactose intolerance, digestive comfort, dairy avoidance, milk intolerance, postprandial

## Abstract

**Background:** Sheep milk (SM) is a possible alternate dairy source for those who experience digestive symptoms with cow milk (CM). While both the milks contain lactose, one of the causes for self-reported intolerance to CM, the composition of SM and CM also differs across proteins and fats, which have been shown to impact digestive processes.

**Objective:** To compare the acute digestive comfort and lactose malabsorption of SM to CM in female dairy avoiders.

**Method:** In a double-blinded, randomized cross over trial, 30 dairy-avoiding females (aged 20–30 years) drank 650 mL of SM or CM (each reconstituted from spray dried powder) following an overnight fast, on two separate occasions at least 1 week apart. Blood samples were collected for glucose and insulin assessment, and single nucleotide polymorphisms of the lactase (*LCT*) gene (C/T_13910_ and G/A_22018_). Breath H_2_ and visual analog scale (VAS) digestive symptom scores were recorded at fasting and regular intervals over 4 h after ingestion.

**Results:** Eighty percentage of study participants were lactase non-persistent (LNP; CC_13910_ and GG_22018_ genotype). Digestive symptoms, including abdominal cramps, distension, rumbling, bloating, belching, diarrhea, flatulence, vomiting, and nausea, were similar in response to SM and CM ingestion (milk × time, *P* > 0.05). Breath H_2_ was greater after CM than SM (72 ± 10 vs. 43 ± 6 ppm at 240 min, *P* < 0.001), which may be due to greater lactose content in CM (33 vs. 25 g). Accordingly, when corrected for the lactose content breath H_2_ did not differ between the two milks. The response remained similar when analyzed in the LNP subset alone (*n* = 20).

**Conclusions:** Despite a higher energy and nutrient content, SM did not increase adverse digestive symptoms after ingestion, relative to CM, although there was a reduced breath H_2_ response, which could be attributed to the lower lactose content in SM. The tolerability of SM should be explored in populations without lactose intolerance for whom underlying trigger for intolerance is unknown.

## Introduction

Dairy is a major source of essential nutrients including Ca (1), high quality proteins, micronutrients (K and Mg), and vitamins (riboflavin, vitamins B_12_, vitamin A, thiamin), in many cultures (1, 2). Complete dairy avoidance may increase the risk of nutrient insufficiency contributing to low bone mineral density, metabolic bone disease, or metabolic syndromes (3, 4). Milk intake has declined over the last few decades, especially in developed countries (5), where adverse gastrointestinal symptoms are a common reason for avoidance (6, 7). This is often attributed to the lactose in cow milk (CM) (8), resulting in lactose malabsorption. Yet, those reporting intolerance to CM are not always diagnosed as lactose intolerant (9, 10), and more recent evidence is emerging that other milk components including the protein fraction may induce similar symptoms (8, 11). However, the majority of people who avoid dairy, do so due to self-reported perception of symptoms rather than a confirmed diagnosis of intolerance (6, 8).

CM is the predominant type of dairy consumed, dominating global milk production (12); however, non-bovine dairy sources have important traditional and cultural origins (13, 14), and are increasing in availability worldwide (15). The increasing awareness of dairy intolerances, cow's milk protein allergy and vegan dietary preferences have all influenced consumers to seek alternative milk substitutes (16). However, segments of the population with substantial nutritional requirements commonly obtained from milk, including infants, children (16), and the elderly (17), may struggle to obtain equivalent nutrient density from plant-based sources (18). Sheep milk (SM) is one alternative to CM, containing higher concentration of micronutrients (Ca and P) (19), and macronutrients (proteins and fats) compared to CM (14, 20). The lactose content in SM and CM do not differ substantially (14) though lactose content in SM may vary with season and lactation period (14, 21). The anecdotal evidence, cited by others, that non-bovine ruminant milks [e.g., goat (22) and sheep (23)] may be better tolerated compared to CM currently lacks clinical evidence.

The compositional along with physiochemical variation between CM and SM may contribute to differences in milk digestion between CM and SM (12, 14, 24, 25). In addition to the higher protein content in SM, the constituent proteins differ between ruminant species. There is a higher β/α_s_-casein ratio in SM (24), compared to CM. This influences the casein micelle formation with higher mineralization and diameter (14) and lower hydration and colloidal stability, resulting in faster coagulation (24), in SM relative to CM. The coagulation of milk has been shown to delay gastric emptying (26), and may contribute to differences in digestion depending on milk composition. Additionally, SM proteins (including caseins) have different sequences than CM proteins (27). This may result in different peptide formation during digestion (28). These differences may have important implications for digestive comfort, as variation in β-casein peptide formation has reported impacts on gastrointestinal transit (6), and may affect lactose digestion and any resulting abdominal discomfort (29–31).

There are limited studies on SM composition and physiochemical properties compared to extensive studies in CM (24). As yet no studies have reported how these differences impact self-reported digestive comfort and lactose malabsorption, particularly in dairy avoiders. Thus, this study aimed to compare the digestive comfort and lactose malabsorption responses to SM in dairy avoiders relative to CM. Due to the compositional and physicochemical differences between SM and CM, we hypothesized that SM would be tolerated better and digested more easily than CM in dairy avoiders including those with lactose intolerance.

## Methods

### Study Design

We conducted a double-blinded, cross over randomized control trial at the Liggins Institute, The University of Auckland between July and November 2018. The primary outcome of the study is reported elsewhere (32). The study was conducted according to the guidelines laid down in the Declaration of Helsinki and all procedures involving human subjects were approved by the New Zealand Health and Disability Ethics Committees (Reference no. 18/NTB/92). The trial was prospectively registered with the Australian New Zealand Clinical Trials Registry (ACTRN12618001030268). Written informed consent was obtained from eligible participants prior to the study commencement.

A total of 32 healthy young women aged 20–40 years with BMI 18–28 kg/m^2^ were recruited using digital and printed advertisements. Two subjects withdrew prior to the completion of the study and were excluded from further analyses (Supplementary Figure 1). All participants self-reported dairy avoidance. Subjects with known dairy allergy, current or history of gastrointestinal, cardiovascular, or metabolic disease, consuming medications expected to interfere with normal digestive and metabolic processes like proton pump inhibitors, antibiotics, or prebiotics (3 months prior to the study) were not eligible.

### Study Procedures

Eligible participants were randomized to consume 650 mL of either SM or CM on two occasions at least 1 week apart. Randomization sequences were computer generated using www.randomizer.org. Both participants and investigators were blinded to the treatment identity and allocation was implemented through sealed envelopes.

Prior to the clinical visits, demographic information was collected, including irritable bowel syndrome (IBS) classification, objectively assessed using Rome III criteria (33). One day prior to the visits, subjects were advised to abstain from vigorous physical exercise, avoid dairy and fiber rich food, and were provided with a standardized low fat, low dietary fiber dinner after which they were to remain fasted from 10.00 p.m.

Upon arrival, fasting breath samples were collected and gastrointestinal symptomology was recorded using a visual analog scale (VAS). A venous cannula was inserted to collect fasting blood samples. Subjects then consumed 650 mL of milk within 10 min and were asked to report their liking and perceived identity of each milk. Following milk ingestion, breath samples were collected every 15 min until 2 h and hourly until 4 h, whereas gastrointestinal symptoms and blood samples were collected every 30 min until 2 h and hourly thereafter until 4 h.

### Digestive Symptoms and Likeability (Visual Analog Scale)

The severity of the subjective digestive symptoms was scored on a 100 mm VAS, with 0 mm corresponding to “no symptoms” and 100 mm corresponding to the “the most severe symptoms imaginable.” The sum of scores for abdominal cramps, rumbling, diarrhea, flatulence, and vomiting >70 out of 500 was indicative of lactose intolerance (34). The other symptoms recorded included abdominal distension, bloating, belching, fecal urgency, digestive comfort, gastric reflux, and nausea.

The likeability of the milk was assessed on a hedonic VAS scale, including taste, aftertaste, smell, visual appeal, and palatability, with “0” mm corresponding to “good” and 100 mm corresponding to “bad.”

### Breath Hydrogen Analyses

AlveoSampler Breath Test Kits were used to collect the breath samples which were then analyzed using a BreathTracker H2+ (Quintron, Milwaukee, WI, USA). Data were recorded as CO_2_ corrected H_2_ concentrations (ppm) as a measure of lactose malabsorption.

### Glucose and Insulin Analyses

Venous blood was collected in EDTA vacutainers (Becton Dickinson & Company, Mount Wellington, New Zealand), and plasma was removed after centrifugation at 2,000 × g for 15 min at 4°C and frozen at −20°C prior to analyses. Plasma glucose and insulin were measured using a Cobas c311 clinical chemistry analyzer (Roche Diagnostics, Manheim, Germany) and Cobas e411 immunoassay analyzer (Roche Diagnostics, Manheim, Germany), respectively.

### Milk Treatments

CM powder was sourced from NZMP (New Zealand Milk Products, Fonterra Co-Operative Group, Auckland, New Zealand). SM powder was sourced from Blue River Dairy (batch no. F2125/HC08) and Spring Sheep Milk Company (batch no. MAN: NOV17-JAN18). Prior to weighing and reconstitution, the SM powders were mixed in a 1:1 ratio. All powders were stored at −20°C until use. The reconstituted SM had higher concentrations of proteins, total solids, total energy, and fats but lower lactose than CM (Table 1). The compositional analyses of the milk were performed by a MilkoScan FT1 (FOSS, Denmark) analyzer using the default milk mosaic software. Additional details of milk composition and analyses are described elsewhere (32).

**Table 1:** Composition of sheep and cow milk (650 mL).

**Component**	**Cow milk**	**Sheep milk**
Total Energy (kJ)	1649.3	2140.4
Fat (g)	21.3	33.4
Protein (g)	19.4	29.9
Lactose (g)	33.3	24.9
Total solids (g)	79.0	91.7

*Compositional analyses of milk was performed using a MilkoScan FT1 (FOSS, Denmark) analyzer. Test drinks were prepared using 81 g of whole cow milk powder or 98 g of whole sheep milk powder, reconstituted in 585 mL of water to make a final volume of 650 mL*.

Spray dried milk powder was reconstituted in water on the day prior to the clinical visit. Pre-weighed portions of CM powder (81 g) or SM powder (98 g) were reconstituted in 585 mL of filtered water heated at 30°C to make a final volume of 650 mL, shaken well and stored at 4°C overnight. The milks were served chilled in a transparent plastic bottle. The reconstitution was performed to match the typical solid content of each milk, with SM having higher solid content than CM.

The volume of 650 mL was chosen to exceed the volume of milk (250 mL) reported to be well-tolerated by those with lactose intolerance (35). This volume is similar to what has been used previously (30, 36–38) to evoke symptoms of digestive discomfort, while providing an appropriate quantity of protein and fat for assessment of the primary and secondary outcomes of the trial (32).

### Lactase Persistence Genotyping

Lactase persistence and lactase non-persistence (LNP) in the study participants was determined using the iPlEX assay and MassARRAY® System (Agena Bioscience, SanDiego, USA) by Grafton Clinical Genomics (GCG, Auckland, New Zealand). Peripheral blood mononuclear cells (PBMCs) were isolated immediately from fasted whole blood collected in EDTA-containing blood collection tubes using a histopaque solution (Sigma- Aldrich, St. Louis, MO, USA) as previously described (39). The samples were stored at −80°C until DNA was extracted. All prep DNA/RNA mini kit (Qiagen, Hilden, Germany) was used to isolate genomic DNA from PBMCs as per the manufacturer's protocol.

### Subset Analyses

Breath H_2_ and sum of symptoms (abdominal cramps, rumbling, diarrhea, flatulence, and vomiting) for lactose intolerance were also analyzed separately in the LNP subset as these individuals are more susceptible to lactose malabsorption and associated symptoms.

### Statistical Analyses

A sample size of 30 was calculated for the primary outcome as described elsewhere (32). However, to provide an 80% power with alpha set at 5%, based on previously provided mean for nausea (8 mm vs. 15 mm) and a standard deviation of 9 mm, 26 subjects would be required (30). The impact of variation in milk composition on digestive symptoms has been previously reported acutely in the context of β-casein variants (30).

Individuals with fasting breath H_2_ above 25 ppm were excluded for breath H_2_ analyses and treated as outliers, as this is identified as the threshold for malabsorption of carbohydrates, and impacts the results of standardized breath hydrogen tests (40). Although best practice for hydrogen breath tests would require rescheduling the test (41), this was not provisioned for in the protocol as lactose malabsorption was a secondary outcome. The primary outcome was to compare the rate of digestibility of the proteins (32) which was not dependent on fasting breath H_2_ below 25 ppm. Values missing completely at random were estimated using multiple imputations as the mean of 5 iterations. The incremental area under the curve (iAUC) was calculated using the trapezoidal method, correcting for baseline concentration. As lactose dose has been shown to affect breath H_2_ concentrations (36, 42, 43), and given the difference in lactose content between the two milks, an adjusted breath H_2_ concentration and iAUC were further calculated and analyzed accordingly to match the lactose content.

iAUC and hedonic likeability were analyzed between the two milks using Student's paired *t*-test. Frequency for identification of milk (CM or SM) was analyzed using Pearson's chi-square test (χ^2^). Other outcomes with multiple factors were compared using repeated measures general linear model with milk and time compared within-subject; multiple comparisons were corrected using Sidak adjustment. Alpha was set at *P* < 0.05 for all tests. All statistical analyses were performed using SPSS version 25 (SPSS, IBM Corporation, Armonk, NY, USA).

## Results

### Demographics

Thirty female participants aged 24.3 ± 1.3 years completed the study with anthropometric and biochemical values within a normal range. Participants self-identified as Caucasian (30%), Asian (37%), and South Asian (33%), with 40% being classified as IBS based on Rome III criteria. Based on lactase genotyping, 80% of the participants were LNP (CC_13910_ and GG_22018_) and only 20% were LP (CT_13910_ or TT_13910_ and GA_22018_ or AA_22018_) (Table 2). Four LNP subjects and one LP subject had a fasting breath H_2_ > 25 ppm and were excluded from malabsorption analyses.

**Table 2:** Baseline characteristics (*n* = 30).

**Measures**	**Values^a^**
Age, y	24.3 ± 1.3
BMI, kg/m^2^	22.8 ± 0.9
Glucose (mmol/L)	4.8 ± 0.1
Insulin (μU/mL)	7.6 ± 0.6
Rome III IBS^b^ *n* (%)	12 (40)
LNP (CC_13910_/GG_22018_)^c^ *n* (%)	24 (80)
Ethnicity	
Caucasian, *n* (%)	9 (30)
Asian, *n* (%)	11 (37)
South Asian, *n* (%)	10 (33)

a *Values presented as mean ± SEM or count (percentage) as indicated*.

b *IBS, Irritable bowel syndrome*.

c *LNP, Lactase non-persistence based on SNPs (single nucleotide polymorphisms) analyses*.

### Milk Likeability and Identification

The frequency of identification reporting indicated that subjects were more likely to perceive CM as CM (*n* = 19) and SM as SM (*n* = 21) (*P* = 0.010, χ^2^). There was no difference in the reported liking between SM and CM for taste, smell, palatability, aftertaste, and visual appeal (*P* > 0.05 each, respectively; Supplementary Table 1).

### Digestive Symptoms in Response to SM and CM

**Figure 1:**
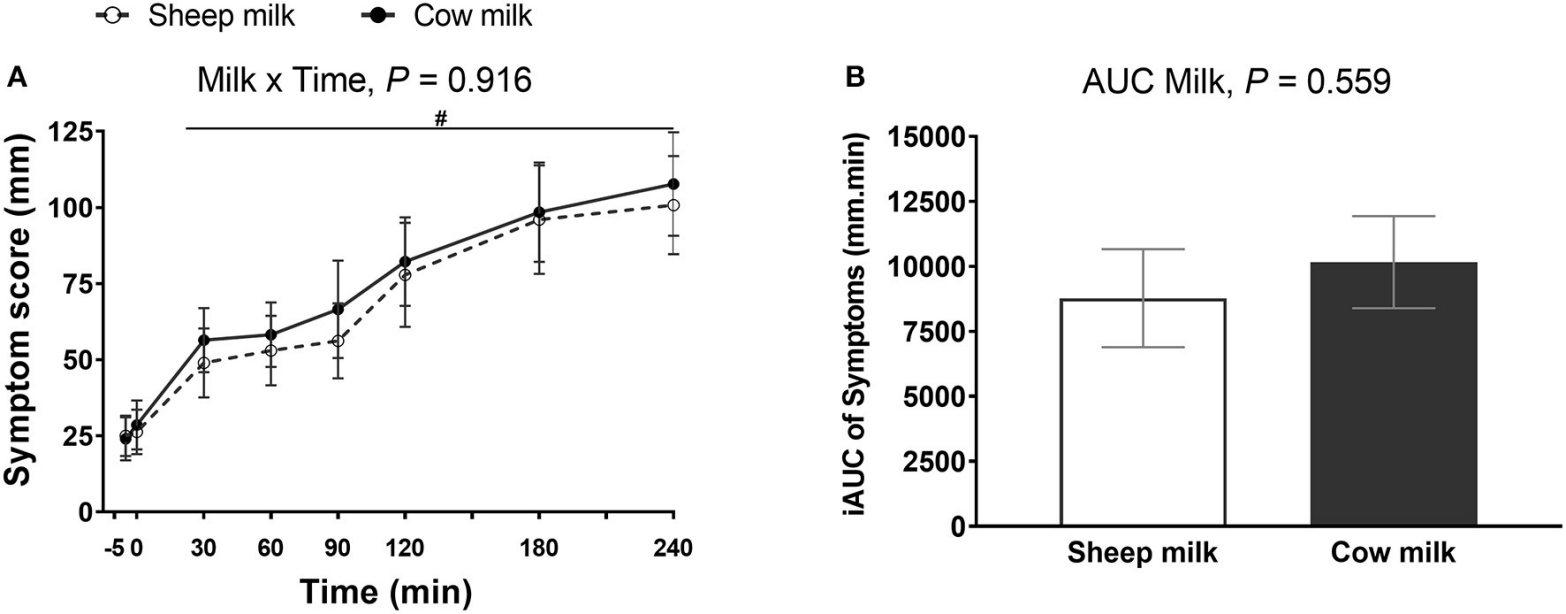
Subjective VAS scores (sum of abdominal cramps, abdominal rumbling, flatulence, diarrhea, and vomiting) **(A)** at multiple timepoints and **(B)** 4-h incremental area under the curve (iAUC) following cow milk and sheep milk ingestion (*n* = 30). Values are presented as means ± SEM. Data for multiple time points were compared using repeated general liner model with milk and time compared within-subject and iAUC was compared using Student's paired *t*-test. There was no milk × time interaction, *P* = 0.916 and iAUC, *P* = 0.559. There was a significant time effect **(A)**, *P* < 0.001. ^#^denotes indicated time points were significantly different from baseline.

There were no differences in the severity of lactose malabsorption associated subjective abdominal symptoms (sum of abdominal rumbling, cramping, flatulence, diarrhea, and vomiting) reported between the two milk types (*n* = 30; milk × time interaction, *P* = 0.916). Likewise, the iAUC did not differ between milks (*P* = 0.559). Regardless of milk type, individuals experienced an increase in lactose malabsorption associated symptoms following milk ingestion (main time effect, *P* < 0.001; Figure 1). Likewise, independent subjective digestive symptoms, including abdominal cramps, rumbling, bloating, belching, flatulence, fecal urgency, diarrhea, nausea, and vomiting, did not differ between milks (milk and time × milk interaction, *P* > 0.05 each, respectively; Supplementary Figure 2) but these symptoms increased following ingestion of either milk (main time effect, *P* < 0.001). No adverse events of vomiting were reported. Gastric reflux was not different from baseline for either milk (main time effect, *P* = 0.305).

### Lactose Malabsorption

**Figure 2:**
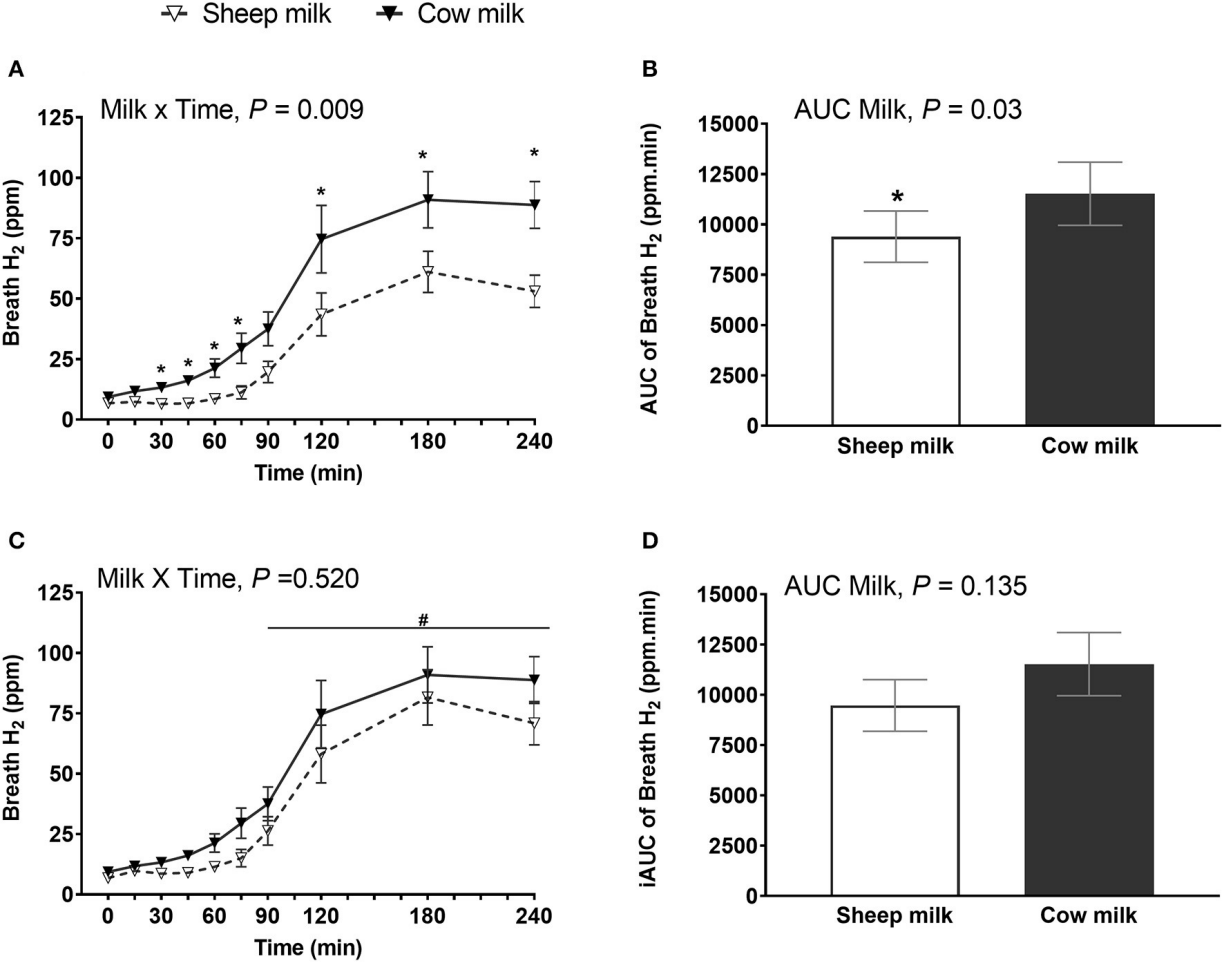
Breath hydrogen following sheep milk and cow milk (*n* = 25, after removal of outliers) at multiple time points **(A,C)** and 4-h incremental iAUC **(B,D)**, before **(A,B)** and after lactose adjustment **(C,D)** i.e., considering equal lactose content in both SM and CM. Values are presented as means ± SEM. Data for multiple time points **(A,C)** was compared by repeated measures general linear model with milk and time compared within-subject and iAUC **(B,D)** was compared using Students paired *t*-test. Prior to lactose adjustment **(A,B)** there was significant milk x time interaction, *P* = 0.013 and iAUC, *P* = 0.015. After lactose adjustment **(C,D)**, there was no milk x time interaction, *P* = 0.069 and iAUC, *P* = 0.131. * denotes *P* < 0.05, indicated timepoints were different between the milks after *post-hoc* correction. and iAUC was different between the milks. There was a significant time effect **(C)**, *P* < 0.001. ^#^denotes indicated time points were significantly different from baseline.

Regardless of milk type, breath H_2_ increased postprandially (*n* = 25; main time effect, *P* < 0.001) but the increment was greater after CM compared to SM (milk × time interaction, *P* = 0.013; *P* < 0.05 *post-hoc* comparison between CM and SM at 30, 45, 60, 75, 180, and 240 min) (Figure 2A). The AUC was also higher after CM compared to SM (*n* = 25; *P* = 0.015) (Figure 2B).

Given the difference in lactose content between the reconstituted milks (33 g in CM vs. 25 g in SM), breath H_2_ was adjusted to match the lactose content. After lactose adjustment (considering the lactose content in SM was 33 g, the H_2_ value for SM was multiplied by 33/25 = 1.32), breath H_2_ did not differ between milks at any time point (milk × time interaction, *P* = 0.069) (Figure 2C), and the iAUC was no longer different between milks (*P* = 0.131) (Figure 2D). However, CM ingestion in general resulted in higher breath H_2_ when corrected for lactose content compared to SM (main milk effect, *P* < 0.001).

### LNP Subset Analysis of Digestive Symptoms and Lactose Malabsorption

Given lactose malabsorption contributes to symptoms of intolerance and is highly linked to LNP status, subset analysis of symptoms and malabsorption were completed on LNP subjects. LNP subjects (*n* = 20) showed the same patterns of response as the total sample set. Digestive symptoms did not differ between milks (*n* = 24; milk × time interaction, *P* = 0.750; iAUC, *P* = 0.365) although increased with time regardless of milk type (main time effect, *P* < 0.001) (Supplementary Figure 3). Breath H_2_ was higher after CM compared to SM (*n* = 20; milk × time interaction, *P* = 0.009 and iAUC, *P* = 0.030) but after lactose adjustment, it did not differ (milk × time interaction, *P* = 0.520 and iAUC, *P* = 0.135; Supplementary Figure 4).

### Plasma Glucose and Insulin Analyses

The plasma glucose and insulin responses did not differ between the milk types. The iAUCs for glucose after ingestion of CM compared to SM were −204 ± 21. vs. −168 ± 23 mmol·min/L, and the iAUCs for insulin were 2,400 ± 186 vs. 2,377 ± 171 μU·min/mL (*P* > 0.05, each, respectively).

## Discussion

SM is a nutritionally rich alternative to CM which may be digested differently, owing to its unique composition and physiochemical properties. Thus, this study investigated digestive comfort and lactose malabsorption following ingestion of SM compared to CM. In contrast to the hypothesis, subjective digestive symptoms did not differ between the two milks, but breath H_2_ was raised to a greater extent by CM than SM. While the dairy avoiders included in this study were largely LNP, indicating likely lactose intolerance, subset analysis of LNP subjects only indicated that the symptoms and lactose malabsorption responses were the same, as for the entire study group.

In these dairy avoiders, ingestion of either milk resulted in increased subjective digestive symptoms. Digestive symptoms reported after milk ingestion are influenced by several factors including compositional variations of milk [lactose (44), fats (45) and protein content (30)], intestinal transit or gastric motility (46), colonic flora (47), and visceral sensitivity (48). Higher fat (45) and energy content (49) are known to slow gastric emptying and fat specifically has been shown to increase the jejunal transit time (50). Variation in milk protein sequences [such as those between SM and CM (28)] and protein integrity [hydrolyzed or whole protein (37)], impact digestive products (28) and gastric emptying (37) or incretin responses (51). Other protein-related effects, such as those observed with A1 β-casein, have also been shown to impact lactose malabsorption and related symptoms (30), however the mechanisms for this are not clear. It is known that delayed intestinal transit of lactose is responsible for reduced malabsorption and symptoms of intolerance, as observed during pregnancy (52). Individual features like visceral hypersensitivity may also induce digestive symptoms in lactose malabsorbers (48), or the onset of the symptoms and severity may depend on the colonic bacteria and their fermentation pathways (47). Although this study did not directly assess gastrointestinal function, the similar subjective digestive symptoms between the milks suggest that in a group that is largely lactose malabsorbers, there may not be large differences.

After equal volumes, breath H_2_ was increased to a greater extent following CM than SM ingestion, indicating higher lactose malabsorption after CM. The present study used reconstituted powdered milk to match the solid content of fresh liquid milk which resulted in a 24% lower lactose content in SM than CM. While some studies report similar lactose content in SM and CM (14, 53), others have reported lower lactose content in SM (54), similar to the content in current study. The composition of SM varies seasonally with lower lactose content at the end of lactation (14, 21). Previous studies show that breath H_2_ may depend on the dose of lactose ingested (55, 56), so the H_2_ concentration was further adjusted to match lactose doses between milks. After the adjustment, overall breath H_2_ (iAUC) was not different between the two milks. It is important to note that although breath H_2_ may depend on the dose of lactose (55, 56), the increment may not be directly correlated (57), especially when milk is used as a substrate (58). The rise in breath H_2_ is influenced by factors including the complexity of the food matrix (36, 58), inter-individual variations, gut microflora (47, 59, 60), and gastrointestinal transit (46). The complex interaction of all these factors precludes calculation of compensatory adjustment of breath H_2_. The adjustment of breath H_2_ concentrations for specific substrates or doses is not a standardized practice (42), and given that lactose dosing may even require adjustment for body weight (61), may not be a straightforward calculation. Although the current study showed lower lactose malabsorption with SM, differences in lactose digestion following SM should be investigated within the upper range of lactose content naturally occurring in SM, which may be more closely matched to CM.

Dairy intolerance is mainly attributed to lactose, with characteristic symptoms of diarrhea, flatulence, abdominal rumbling, cramping, and vomiting (34, 44). Despite lower lactose content of SM resulting in lower lactose malabsorption, no impact on subjective digestive comfort was observed. This supports evidence in the literature showing that although lactose malabsorption may result in abdominal discomfort (62), the severity of digestive symptoms is not always correlated to lactose malabsorption (57). As such, lowering the lactose content in milk may improve lactose malabsorption but not tolerance. However, the majority of subjects in the current study were LNP as defined by *LCT* gene SNPs C/T_13910_ and G/A_22018_, and were lactose malabsorbers as reflected in their breath H_2_ concentrations. Most LNP individuals tolerate low doses of lactose (<12 g per serving) (44, 62) but above this dose report symptoms. As this study used a high volume of milk (650 mL), the lactose content was sufficient (>12 g) to induce symptoms in lactose intolerant individuals despite the compositional variations. However, it should be noted that such a high volume is more than twice a usual serving size of milk—this high volume of milk may have resulted in more severe symptoms than might be expected for those with sensitivity to milk. For SM with a higher nutrient density, this effect may have been greater. It is unclear whether a more usual serving size may have revealed differences in the severity of subjective symptoms between SM and CM. Indeed, the underlying intolerance explains the prevalence of symptoms associated with lactose intolerance reported following ingestion of both milks. Furthermore, these symptom and breath H_2_ findings remained the same when analyzed with LNP subset, highlighting that the current study is mainly reflective of lactose mediated intolerance, but may not be representative of SM tolerance with a non-lactose mediated dairy intolerance. Thus, further studies are required to determine whether individuals without lactose malabsorption experience different digestive tolerance to SM relative to CM, and whether typical serving sizes of SM are tolerated differently to CM.

Dairy intolerance has been reported to be caused by characteristics of milk other than lactose. Intolerance can occur in the absence of lactose malabsorption (30, 63) and has been attributed to sensitivity to bioactive peptides released during milk digestion (64). In this case, it may have been expected that the lack of some milk proteins, like A1 β-casein in sheep milk (27), may have contributed to less discomfort than CM, as A1 β-casein has been implicated in digestive discomfort (64). Yet, the proportion of A1 β-casein in the CM may have been less than expected, as New Zealand conventional bovine herds have been reported to produce milk with only ~22% A1 β-casein (30), and may in part explain a lack of difference in subjective symptoms. Indeed, the variety of bioactive peptides in milk, many with known links to IgE and non-IgE-mediated immune responses (65), could have contributed to symptoms in the current study. Although those with known milk allergy were excluded, no comprehensive testing of milk protein sensitivity was conducted. Differing or modification of dairy structures are known to influence digestion and resulting physiological responses (66); homogenization and pasteurization (impacting protein and fat structures), has been reported to aggravate intolerance symptoms, particularly in those with lactose malabsorption (38). Therefore, species-specific physiochemical differences independent of lactose, including density, may have influenced tolerance to SM and CM in the current study. Further, the large variability in symptom responses across subjects highlights the diverse experience of “dairy avoiders” in response to milk. This aligns with the recent description of differing symptoms traits and severity in differing types of dairy intolerance (30), and suggests that detection of differences in comparative symptoms between SM and CM may be more clear in specific subsets of dairy intolerance (e.g., not lactose intolerant) with a common pathophysiology Besides, digestive comfort and lactose malabsorption, SM provides more branched chain amino acids which may benefit individuals with increased protein requirements (32).

The current study included only young female participants, who were largely lactose intolerant, and may not represent all people who avoid bovine dairy or seek alternatives. The prevalence rate of gastrointestinal symptoms (67) and IBS (68) are higher in females than males which may suggest gastrointestinal symptoms reported in the present study could be overestimated. In addition, age related physiological changes in the gastrointestinal tract (69) may impact the digestive process and digestive symptoms in the elderly. Thus, generalizability of these findings to males, older populations, or tolerant individuals is limited, warranting further studies considering both sexes of different age groups and including a control dairy-free comparator. The pain tolerance in females may also vary with the stage of the menstrual cycle (70) and with a wash out of only 1 week, which given the possibility of a relationship between menstrual phase and digestive symptoms, this may have impacted on the symptoms reported (71). However, this was not considered in the present study. Furthermore, despite a trend of a higher digestive symptoms after CM compared to SM, these differences were not significant. In part this may be due to the relatively small sample size and the wide variations of self-reported subjective symptoms experienced. These findings highlight the need to better understand the spectrum of intolerance pathophysiology in non-lactose dairy intolerant individuals.

Perceptual differences between milks may have also mediated subjective symptom reports in this study. Milks were blinded but not masked, and participants were able to differentiate SM and CM by taste. However, as this had no impact on likeability scores, there may not have been a notable influence on symptoms. Further, although VAS are validated for pain (72), these scales may not have captured symptoms of discrete events, like vomiting and diarrhea, adequately. In the current study, no adverse events of vomiting were recorded, yet reports by VAS showed high variability between subjects—possibly reflecting associated feelings, rather than a discrete event. As such, these symptoms may be poorly suited for timed VAS reporting, reflected in the high variability between subjects. This is supported by literature showing that feelings of nausea, rather than vomiting itself, are validated by VAS (73). This study only explored acute digestive responses. Longer term studies may show different kinetics, as habitual dairy ingestion may improve tolerance to lactose (74). Furthermore, the compositional discrepancies across macro (44, 45) and micronutrient contents (75) in the reconstituted milk relative to their naturally occurring counterparts may have influenced digestion. Despite the variation in dairy both seasonally and across species/products (14), the current study serves to provide initial insights into acute digestive response following an equal volume of SM compared to CM.

In summary, dairy avoiders, who were largely intolerant to lactose in CM, experienced similar digestive symptoms following an equal volume of SM and CM, despite higher lactose malabsorption after CM ingestion. This highlights that the digestive discomfort of milk intolerance is complex and impacted by more than just lactose. Further, tolerability of SM over CM should be additionally explored in populations without lactose intolerance but who still experience adverse symptoms associated with milk ingestion, for whom the underlying trigger of intolerance may be unclear.

## Data Availability Statement

The datasets generated for this article are not readily available because approval has not been granted by subjects. Requests to access the datasets should be directed to a.milan@auckland.ac.nz.

## Ethics Statement

The studies involving human participants were reviewed and approved by New Zealand Health and Disability Ethics Committees. The patients/participants provided their written informed consent to participate in this study.

## Author Contributions

AS conducted research, analyzed data, performed statistical analyses, and wrote the first draft of the paper. LS, LD, and DC-S designed research and reviewed the paper. PS conducted research. AM designed and conducted research, wrote the paper, and had primary responsibility for final content. All authors approved the final version of the manuscript for submission.

## Conflict of Interest

AM, LS, and LD are current employees of AgResearch Limited. The remaining authors declare that the research was conducted in the absence of any commercial or financial relationships that could be construed as a potential conflict of interest.
