# Supplementary material for: Comparing Response of Sheep and Cow Milk on Acute Digestive Comfort and Lactose Malabsorption: A Randomized Controlled Trial in Female Dairy Avoiders
Source: Front Nutr. 2021 Feb 15;8:603816. doi: 10.3389/fnut.2021.603816 (PMC7917135; doi:10.3389/fnut.2021.603816)
Supplement: Supplementary file 1 [file Data_Sheet_1.pdf]

Supplementary Material

1 Supplementary Table

Table 1. **The hedonic attribute scores for sheep milk and cow milk**

Hedonic attribute	Sheep milk[1]	Cow Milk	*P* value[2]
Aftertaste	50.6 ± 6	43.4 ± 5.6	0.357
Palatability	51.5 ± 6.4	38 ± 4.9	0.061
Smell	48.7 ± 5.6	53 ± 5.8	0.298
Taste	48.1 ± 6.1	38.7 ± 5.5	0.185
Visual appeal	33.1 ± 5.7	37.2 ± 5.8	0.309

[1]Values presented as mean ± SEM in mm, with “0” mm corresponding to “good” and 100 mm corresponding to “bad”.

[2]Significance determined using Student’s paired *t*-test.

2 Supplementary Figure

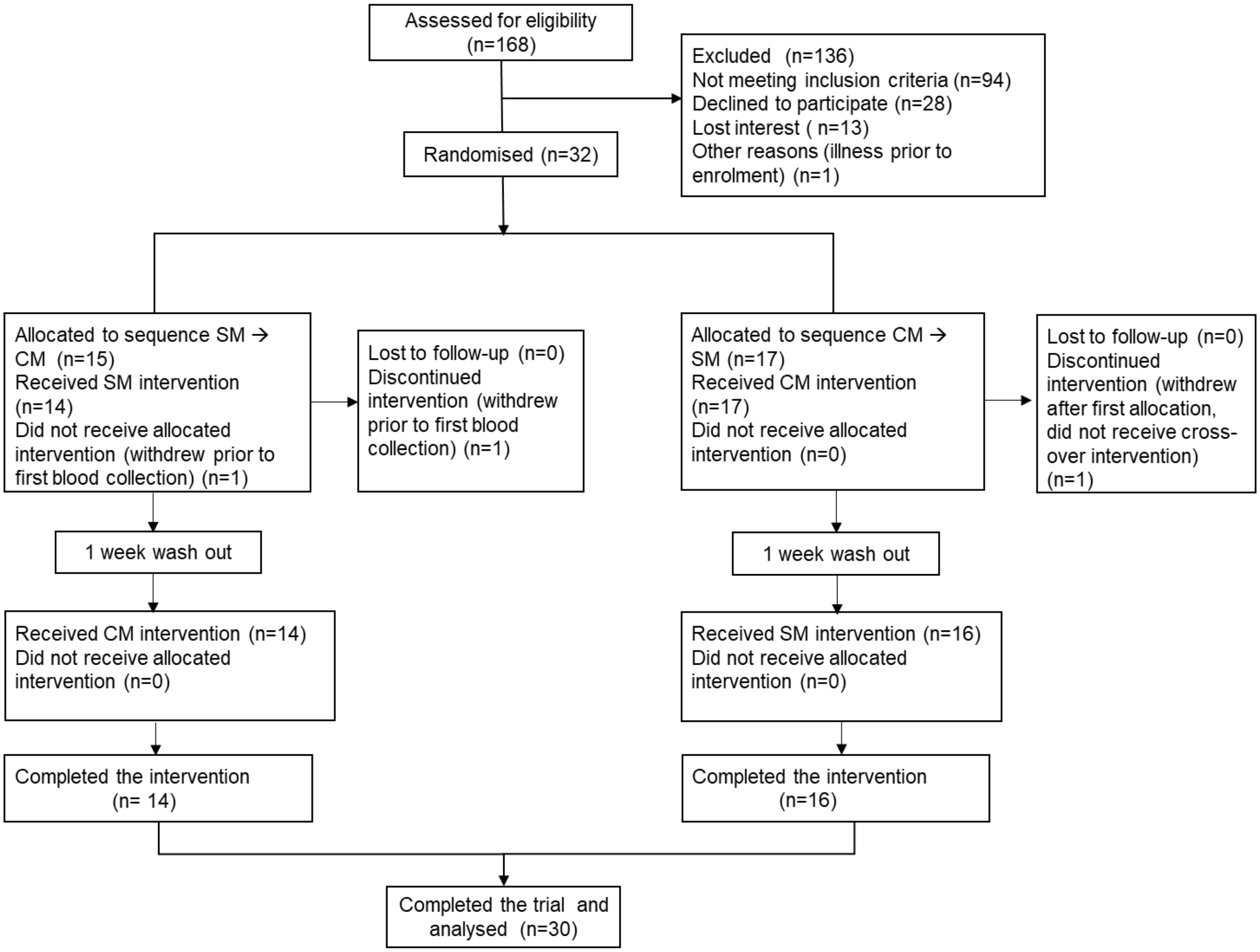

Supplementary Figure 1. Consort flow diagram of study participant recruitment, intervention, follow up and analysis. SM: Sheep milk and CM: Cow milk.

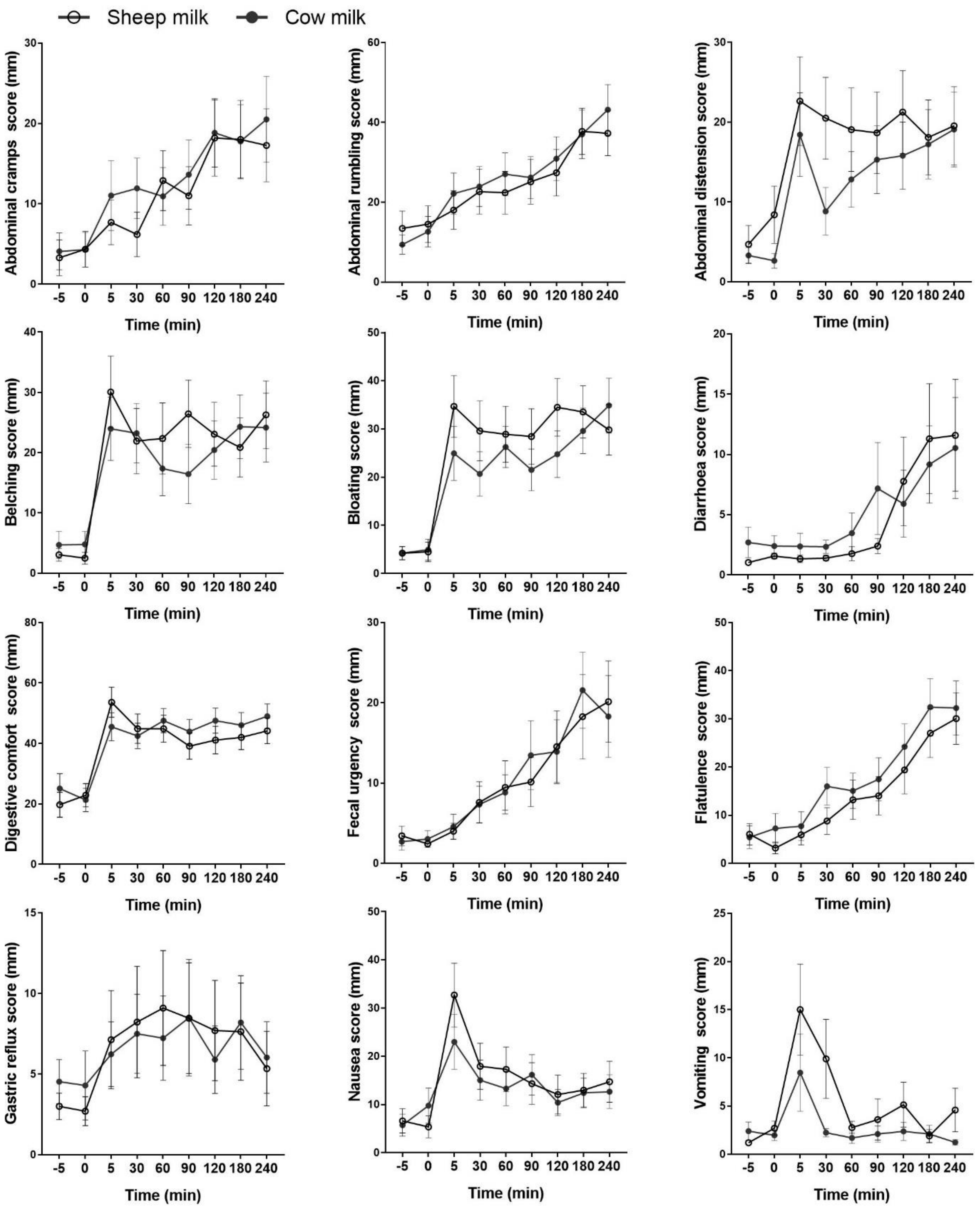

Supplementary Figure 2. Digestive symptoms as measured by VAS score at multiple time points following cow milk and sheep milk ingestion (n = 30). (A) abdominal cramps, (B) abdominal rumbling, (C) fecal urgency, (D) diarrhoea, (E) vomiting, (F) flatulence, (G) digestive discomfort, (H) nausea, (I) belching, (J) abdominal distension, (K) belching, and (L) gastric reflux. Values are presented as means ± SEM. No adverse events of vomiting were reported; although vomiting was reported on a spectrum, score more likely reflect feelings of vomiting rather than a discrete event, supported by the large error bars. Data were compared using repeated general liner model with milk and time compared within-subject. There was no milk × time interaction for any of the symptoms, $P > 0.05$ for each respectively.

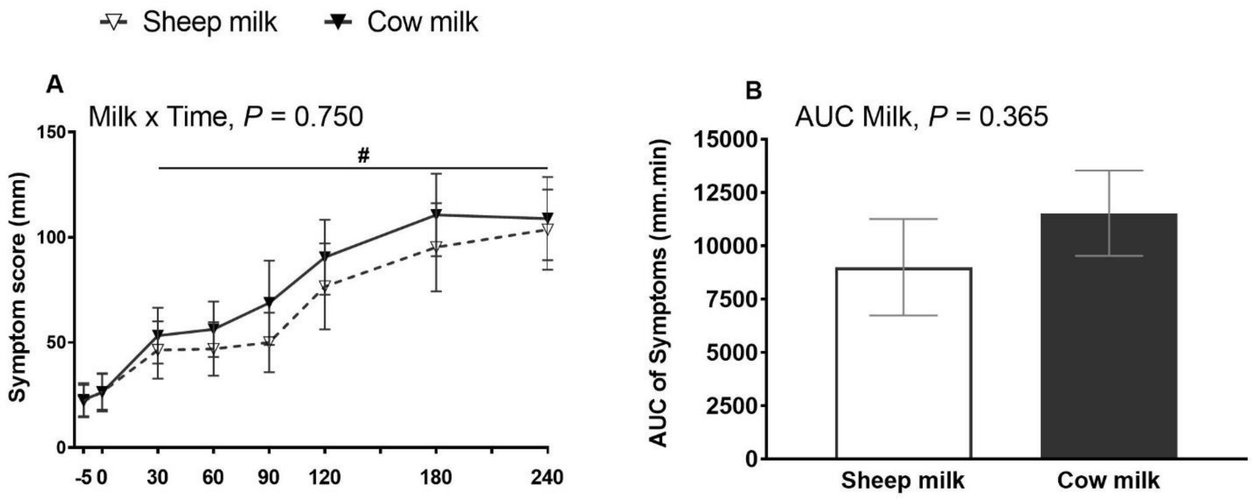

Supplementary Figure 3. Subjective VAS scores (sum of abdominal cramps, abdominal rumbling, flatulence, diarrhoea and, vomiting) in LNP subset (n = 24). A) at multiple time points and B) four-hour incremental area under the curve (AUC) following cow milk and sheep milk ingestion. Values are presented as means ± SEM. Data for multiple time points were compared using repeated general liner model with milk and time compared within-subject. AUC was compared using Students paired *t*-test. There was no milk × time interaction, $P = 0.750$ and AUC, $P = 0.365$. There was a significant time effect (A), $P < 0.001$. # denotes indicated time points were significantly different from baseline.

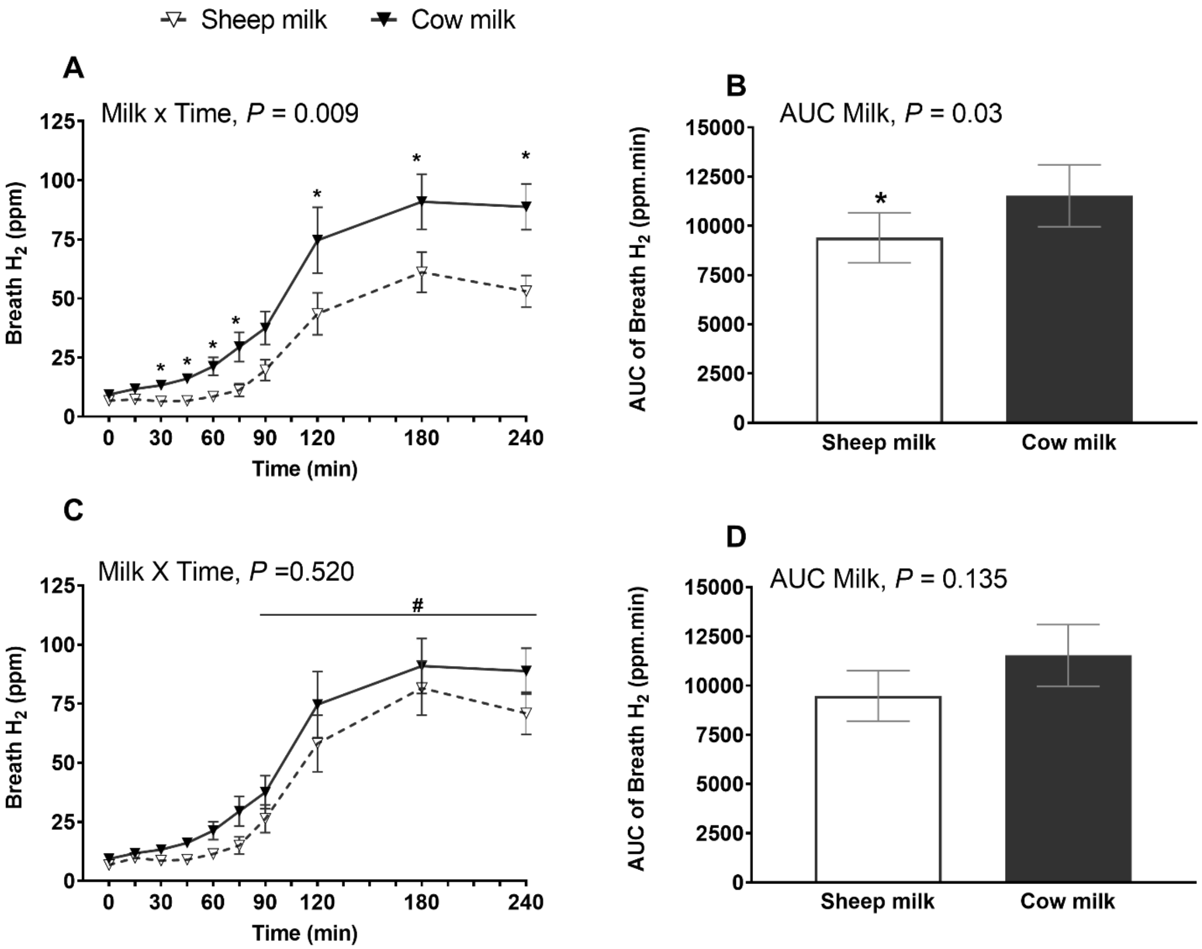

Supplementary Figure 4. Breath hydrogen following sheep milk and cow milk ingestion in the LNP subset (n=20, after removal of the outliers) at multiple time points (A, C) and four-hour incremental AUC (B, D), before (A, B) and after lactose adjustment (C, D).Values are presented as means ± SEM. Data for multiple time points (A, C) was compared by repeated measures general linear model. Prior to lactose adjustment (A, B) there was significant milk x time interaction, P = 0.009 and AUC, P = 0.030. After lactose adjustment (C, D), there was no milk × time interaction, P = 0.520 and AUC, P = 0. 135. * denotes $P < 0.05$, indicated timepoints were different between the milk after post hoc correction and AUC was different between the milks. There was a significant time effect (C), $P < 0.001$. # denotes indicated time points were significantly different from baseline.
